# Hepatitis B vaccination coverage among health care workers in China

**DOI:** 10.1371/journal.pone.0216598

**Published:** 2019-05-07

**Authors:** Qianli Yuan, Fuzhen Wang, Hui Zheng, Guomin Zhang, Ning Miao, Xiaojin Sun, Joseph Woodring, Po-lin Chan, Fuqiang Cui

**Affiliations:** 1 National Immunization Program, Chinese Center for Disease Control and Prevention, Beijing, China; 2 Beijing Center for Disease Control and Prevention, Beijing, China; 3 World Health Organization, West Pacific Region Office, Manila, Philippines; 4 Department of Laboratorial Science and Technology & Vaccine Research Center, School of Public Health, Peking University, Beijing, China; Centre de Recherche en Cancerologie de Lyon, FRANCE

## Abstract

**Objectives:**

Nation-wide hepatitis B vaccination coverage among healthcare workers (HCWs) is not well researched in China. This study aims to investigate the self-reported hepatitis B vaccination status among HCWs in China.

**Methods:**

We conducted a cross-sectional survey of health_care workers’ vaccination statuses in 120 hospitals in China by collecting demographic and vaccination data. Univariate and multivariate logistic regression analysis were used to assess factors associated with hepatitis B vaccination coverage.

**Results:**

Eighty-six percent (2,666/3,104) of respondents reported having received at least one dose of the hepatitis B vaccination and 60% (1,853/3,104) reported having completed ≥3 doses of the hepatitis B vaccination. Factors associated with completing ≥3 doses of the hepatitis B vaccination included workplaces offering free hepatitis B vaccination with vaccination management, age, medical occupation, hospital level, acceptable hepatitis B knowledge and having received training on hepatitis B. HCWs in workplaces offering a free hepatitis B vaccine with vaccination management were 1.4 times more likely (OR = 1.4, *95% CI*: 1.1–1.8) to complete their hepatitis B vaccination compared to HCWs in workplaces that did not offer a free hepatitis B vaccine. Either the possession of acceptable hepatitis B knowledge or an age of 30–39 years increased the odds of complete hepatitis B vaccination by 1.3-fold (*95% CIs*: 1.1–1.5 and 1.1–1.7, respectively) over their referent category. The receipt of training on hepatitis B was also associated with a higher percentage of completing the hepatitis B vaccination (OR = 1.5, *95% CI*: 1.2–1.8). The main self-reported reason for incomplete hepatitis B vaccination was “forgot to complete follow-up doses” among 43% (234/547) of respondents. Among those who never received any hepatitis B vaccination, only 30% (131/438) intended to be vaccinated. Obtaining immunity from work (40%) and hospitals that did not provide hepatitis B vaccination activities (40%) were the top reasons mentioned for refusing hepatitis B vaccination.

**Conclusions:**

The complete hepatitis B vaccination rate among HCWs in China is low, and the desire of HCWs for vaccination is indifferent; therefore, education campaigns are needed. In addition, a free national hepatitis B vaccination policy for HCWs that includes vaccination management should be prioritized to improve hepatitis B coverage among HCWs who are at-risk for HBV infection.

## Introduction

Hepatitis B virus (HBV) infection is a major global health problem, with an estimated 257 million people infected with HBV worldwide in 2015 [1]. Approximately 887,000 deaths in 2015 were associated with two main HBV-related complications: cirrhosis and hepatocellular carcinoma [1]. In China, the prevalence of hepatitis B in the general population is estimated to be 5.5% [2], which classifies China as a country with an intermediate endemicity [3]. Hepatitis B vaccination was originally introduced into China’s national children immunization program in 1992. As a result, the HBsAg prevalence rate has significantly decreased in the population aged less than 5 years from 9.67% in 1992 to 0.32% in 2014 [4, 5]. The current hepatitis B vaccination schedule in China is a dose administered within 24 hours of birth, followed by two additional doses administered at 1 and 6 months. However, HBV infection remains a major public health challenge among Chinese adults born before the introduction of the hepatitis B vaccine. Adults born before 1992 were recently shown to have the highest HBV prevalence, with an adjusted relative risk of 4.4 (95% CI: 3.1–6.2) compared to individuals born after 1992 [6].

The World Health Organization (WHO) recommends the hepatitis B vaccine for adults at the highest risk of acquiring HBV infection, which include patients who frequently require blood or blood products, persons who use injecting drugs, household and sexual contacts with persons with a chronic HBV infection, persons with multiple sexual partners and healthcare workers (HCWs) [7]. In addition, the WHO set milestones that over 80% of countries in the Western Pacific Region develop a national policy of vaccinating HCWs against HBV infection by 2017 and that all countries develop a policy by 2020 [8]. In September 2017, the São Paulo Declaration on Hepatitis was launched at the World Hepatitis Summit 2017, calling upon governments to include hepatitis B vaccines for HCWs in national immunization programs [9]. Hepatitis B vaccination is now mandatory for all HCWs in Australia, Austria, Belgium, Canada, the Czech Republic, Germany, Greece, Holland, Ireland, Italy, Poland, Slovakia, Sweden and the United States, as well as for specific HCWs in direct contact with patients or body fluids in France [10–15]. A national policy does not currently exist in China, although some hospitals have developed their own hepatitis B screening and vaccination policies for HCWs.

Hepatitis B infection is an important occupational hazard for HCWs who have the potential for exposure to patients and/or infectious materials, including bodily fluids such as blood, semen and vaginal secretions, contaminated medical supplies and equipment and contaminated environmental surfaces [16]. The annual estimated proportion of HCWs exposed to HBV globally is 5.9%, which corresponds to an estimated 66,000 preventable HBV infections each year among HCWs worldwide [17]. The risk of HBV infection in an unvaccinated person from a single HBV-infected needle stick injury ranges from 6–30% [18, 19]. Hepatitis B vaccination is the most effective method to prevent HBV infection. The achievement and maintenance of a high HCW vaccination coverage level reduces HBV transmission to and from HCWs and their patients [13, 20].

China currently lacks a nation-wide vaccination policy for HCWs, with limited data available on vaccination coverage among HCWs. Existing studies reported different findings on the hepatitis B vaccination status, ranging from 42.4% to 86.4% hepatitis B vaccination coverage among HCWs [21–23]. Moreover, these existing studies are limited in terms of geographical coverage, primarily covering tertiary hospitals located in large cities. Therefore, this study estimates hepatitis B vaccination coverage and the associated factors among HCWs in China. Findings are expected to provide further evidence that may inform policies on hepatitis B vaccination strategies for HCWs in China.

## Materials and methods

The Fujian, Jiangxi and Gansu Provinces were chosen to sample HCWs, as these provinces represent eastern, central and western China respectively. Cities in each province were divided into two groups according to HCW hepatitis B vaccination policies. In each group, municipal-, county- and township-level hospitals were selected using a convenient sampling method. Departments were categorized into two groups according to their probabilities of exposure to blood or bodily fluids from patients, based on previously published data [15,23–26]. High-risk departments include the operation room, surgery, gynecology and obstetrics, anesthetic room, haemodialysis, laboratory for blood or body fluid chemistry, blood transfusion room, housekeeping services and dental services. Low-risk departments include the pharmacy room, x-ray rooms, electrocardiogram room, ultrasound room, and administrative services.

HCWs born before 1992 with over six months of job experience were selected using a convenient sampling method. Interns and medical students were excluded from the analysis. HCWs included physicians (such as surgeons, internist physicians and dentists) who were involved in providing direct health care services to patients, nurses, technicians, pharmacists, laboratory personnel, cleaners and administrative officers. Because the pharmacy room and medical laboratory room belong to the “Medical Technology Department” in China, HCWs working as technicians, pharmacists and laboratory personnel were classified into the “medical technician” category in the final analysis. Demographic information, hepatitis B knowledge, awareness of the current HBV infection status, hepatitis B vaccination history and reasons for vaccine refusal were collected through a self-administered questionnaire and an anonymous survey.

### Ethical review

This study was approved by the Chinese Center for Disease Control and Prevention Ethical Review Committee. Prior to enrolment, the selected HCWs were informed of the study aims. Once the participants agreed to participate and oral informed consent was obtained, a face-to-face interview was conducted by trained interviewers.

### Definitions

#### Vaccination management among HCWs

Hospitals were categorized into having 1) no availability of a free hepatitis B vaccine; 2) a free and available hepatitis B vaccine without a HCW vaccination management program; or 3) a free and available hepatitis B vaccine with a HCW vaccination management program. A HCW vaccination management program refers to the presence of a vaccine uptake surveillance system that includes anti-HBs status guidance and re-vaccination assessment.

#### Hepatitis B knowledge

Hepatitis B knowledge was assessed using a questionnaire that included seven yes/no/unknown questions and two multiple choice questions. One point was awarded for each correct answer and no points were awarded for an incorrect response, with a resulting total score ranging from 0–14 points. An overall hepatitis B knowledge score was categorized as either having “adequate knowledge” or “inadequate knowledge” based on whether an individual’s score was above or below the mean score [27–29].

#### At least one dose of vaccination or complete vaccination rate

Respondents were asked the following questions: “Have you ever received a hepatitis B vaccination (yes/no/unknown)?”; those responding “yes” were then asked, “How many doses have you received?” According to the number of doses they received, HCWs were categorized as having received either no hepatitis B vaccination dose, at least one dose (≥1 dose) or ≥3 doses. Self-reported HBV infection information among those participants who knew their vaccination status was also collected.

### Statistical analysis

Data were entered into Epi Data version 3.1 software (Epi Data Association, Odense, Denmark). Statistical analyses were performed using SPSS version 21 (SPSS, Chicago, IL, USA). Outcome variables were “at least one dose of hepatitis B vaccination rate” and “complete hepatitis B (≥3 doses) vaccination rate”. Predictor variables included age, gender, years working in the profession, education, medical occupation, department, hospital level, province, free hepatitis B vaccination program in workplace, HBV-related training and knowledge. Associations between predictor variables and outcomes were estimated using both univariate and multivariate logistic regression analyses. We entered variables that attained a significance level of *p* < 0.1 in the univariate analysis into the multivariable model, as well as variables that were considered potential confounders, regardless of their level of statistical association in the univariate analysis. We used a multivariate regression analysis with stepwise backward variable selection to test for factors predicting vaccination coverage, with significance level of selection entry at 0.05 and selection of stay at 0.10.

## Results

### Basic characteristics of the study population

In this study, 120 hospitals were surveyed, including 11 municipal hospitals, 17 county hospitals and 92 township hospitals. Among 4,850 HCWs investigated, 417 (9%) HCWs refused to participate, 171 (4%) participants were ineligible because they were born after 1992, and 94 (2%) HCWs submitted incomplete questionnaires. The analysis included 4,168 (86%) eligible HCWs who completed questionnaires.

**Table 1** summarizes the demographic information of the interviewed HCWs. Of these 4,168 HCWs, 36% were from township-level hospitals and 33.0% were from county–level hospitals. In addition, 60% of HCWs worked in low-risk departments, while 40% worked in high-risk departments. The male to female ratio was 1:2. Ages ranged from 25–60 years, with a median of 35.5±8.7 years. Physicians (34%) and nurses (32%) accounted for the majority of HCWs.

**Table 1:** Characteristics of HCWs enrolled and mean score distribution of hepatitis B knowledge (n = 4,168).

Features	No. of HCWs	Proportion (%)	Mean score
**Province**			
Fujian	913	22	10.7
Jiangxi	1,711	41	10.2
Gansu	1,544	37	9.9
**Hospital level**			
Municipal	1,306	31	9.9
County	1,376	33	10.2
Township	1,486	36	10.4
**Gender**			
Male	1,421	34	10.2
Female	2,747	66	10.2
**Age group, years**			
25–29	1,301	31	10.1
30–39	1,610	39	10.4
40 or more	1,257	30	10.1
**Number of years working in the profession**			
1–4	822	20	10.0
5–14	1,830	44	10.2
15 or more	1,516	36	10.2
**Education**			
Junior college or lower	1,842	44	9.9
Undergraduate	2,090	50	10.4
Postgraduate and higher	236	6	10.5
**Medical occupation**			
Physician	1,424	34	10.4
Nurse	1,342	32	10.2
Technician	828	20	10.0
Administrator	515	12	10.2
Cleaner	59	1	9.0
**Department**			
Low-risk	2,492	60	10.2
High-risk	1,676	40	10.1
**Workplace offers free hepatitis B vaccination**			
No	2,097	50	10.1
Yes, without management	1,184	28	10.0
Yes, with management	887	21	10.6
Total	4,168	100	10.2

### Hepatitis B vaccination coverage

Among the 4,168 HCWs, 6% (256/4,168) did not know their hepatitis B vaccination history. Seventy-nine percent (3,277/4,168) reported that they were vaccinated and 15% (635/4,168) reported that they did not receive the hepatitis B vaccination. Hepatitis B vaccination coverage were analysed among HCWs with a known vaccination history (vaccinated or unvaccinated), and the self-reported HBsAg tests were negative (n = 3,104). Of these 3,104 HCWs, 86% (2,666/3,104) stated that they received at least one dose of the hepatitis B vaccine and 60% (1,853/3,104) reported that they were completely vaccinated.

### At least one dose of the hepatitis B vaccine

According to the results of the multivariate logistic regression analysis, the workplace that offers free hepatitis B vaccination with vaccination management, age group, medical occupation, department, and having received hepatitis B training were factors associated with the receipt of ≥1 dose of the hepatitis B vaccine (Table 2). Compared to HCWs in workplaces that do not offer a free hepatitis B vaccine, HCWs in workplaces offering a free hepatitis B vaccine with vaccination management had a 2.7 times greater odds ratio of ≥1 hepatitis B vaccination (*95% CI*: 1.9–3.9). HCWs who received hepatitis B training had a 2.0 times greater odds ratio of receiving ≥1 hepatitis B vaccination compared to HCWs who had not received hepatitis B training (*95% CI*: 1.6–2.4). Compared to HCWs 40 years or older, HCWs aged 25–29 years (OR = 2.0, *95% CI*: 1.2–3.3) and HCWs aged 30–39 years (OR = 2.5; *95% CI*: 1.7–3.7) were more likely to have received ≥1 hepatitis B vaccination. Working in a low-risk department was associated with a higher odds ratio for having received ≥1 hepatitis B vaccination (OR = 1.5, 95% CI: 1.2–1.9).

**Table 2:** Multivariate analysis of the hepatitis B vaccination coverage rate among HCWs in China (n = 3,104).

Variables	Total No.	No. vaccinated (%)	No. completely vaccinated (%)	Multivariate analysis			
				≥1 dose		≥3 doses	
				OR (95% *CI*)	*P*	OR (95% *CI*)	*P*
**Province**							
Fujian	681	557 (82)	368 (54.0)	Ref		Ref	
Jiangxi	1,223	1,018 (83)	678 (55.4)	1.2 (0.9–1.5)	0.22	1.1 (0.9–1.3)	0.63
Gansu	1,200	1,091 (91)	807 (67.3)	1.5 (1.0–2.0)	0.04	1.5 (1.2–2.0)	<0.01
**Hospital level**							
Municipal	908	789 (87)	473 (52.1)	Ref	Ref	Ref	
County	1,035	874 (84)	612 (59.1)	0.9 (0.6–1.2)	0.35	1.3 (1.1–1.6)	<0.01
Township	1,161	1,003 (86)	768 (66.2)	0.9 (0.7–1.2)	0.61	1.6 (1.4–2.0)	<0.01
**Workplace offers free hepatitis B vaccination**							
No	1,525	1,281 (84)	848 (55.6)	Ref		Ref	
Yes, without management	876	721 (82)	500 (57.1)	0.9 (0.7–1.1)	0.21	1.0 (0.8–1.2)	0.88
Yes, with management	703	664 (94)	505 (71.8)	2.7 (1.9–3.9)	<0.01	1.4 (1.1–1.8)	0.02
**Gender**						-	
Male	1,048	875 (84)	626 (59.7)	Ref		Ref	
Female	2,056	1,791 (87)	1227 (59.7)	1.1 (0.9–1.4)	0.41	1.0 (0.8–1.2)	0.67
**Age group**							
25–29	937	834 (89)	519 (55.4)	2.0 (1.2–3.3)	<0.01	1.0 (0.7–1.4)	0.80
30–39	1,203	1,072 (89)	750 (62.3)	2.5 (1.7–3.7)	<0.01	1.3 (1.1–1.7)	0.04
40 or older	964	760 (79)	584 (60.6)	Ref		Ref	
**Number of years working in their profession**							
1–4	582	527 (90)	331 (56.9)	1.3 (0.8–2.2) 90	0.36	Ref	
5–14	1,360	1,194 (88)	797 (58.6)	0.8 (0.6–1.2)	0.38	0.8 (0.6–1.0)	0.11
15 or longer	1,162	945 (81)	725 (62.4)	Ref		1.1 (1.1–0.8)	0.51
**Medical occupation**							
Physician	1,093	912 (83)	621 (56.8)	Ref		Ref	
Nurse	975	866 (89)	597 (61.2)	1.4 (1.1–1.8)	0.01	1.3 (1.1–1.6)	0.01
Medical technicians	631	535 (85)	373 (59.1)	1.2 (0.9–1.6)	0.13	1.2 (1.1–1.5)	0.04
Administrator	371	324 (87)	241 (65.0)	1.2 (0.8–1.7)	0.37	1.2 (0.9–1.5)	0.18
cleaner	34	29 (85)	21 (61.8)	2.1(0.8–5.5)	0.16	1.5 (0.7–3.1)	0.29
**Department**							
Low-risk	1,858	1,632 (88)	1,159 (62.4)	1.5 (1.2–1.9)	<0.01	Ref	
High-risk	1,246	1,034 (83)	694 (55.7)	Ref		1.2 (0.99–1.4)	0.07
**Hepatitis B training**							
No	812	642 (79)	412 (50.7)	Ref		Ref	
Yes	2,292	2,024 (88)	1,441 (62.9)	2.0 (1.6–2.4)	<0.01	1.5 (1.2–1.8)	<0.01
**Hepatitis B knowledge**							
Unacceptable	1,702	1,443 (85)	953 (56.0)	Ref		Ref	
Acceptable	1,402	1,223 (87)	900 (64.2)	1.1 (0.9–1.4)	0.41	1.3 (1.1–1.5)	<0.01
**Total**	3,104	2,666 (86) )	1,853 (59.7)	0.02	1.42	<0.01	0.44

### Complete hepatitis B vaccination rate

Compared to HCWs in workplaces that do not offer a free hepatitis B vaccine, HCWs in workplaces offering a free hepatitis B vaccine with vaccination management had a 1.4 times higher odds ratio of complete hepatitis B vaccination (*95% CI*: 1.0–1.8). The possession of acceptable hepatitis B knowledge or an age of 30–39 years increased the odds ratio of complete hepatitis B vaccination approximately 1.3 times compared with the reference category (*95% CI*: 1.1–1.7). Having received hepatitis B training was also associated with a higher complete hepatitis B vaccination rate (OR = 1.5, *95% CI*: 1.2–1.8). Compared to physicians, medical technicians (OR = 1.2, *95% C*I: 1.1–1.5) and nurses (OR = 1.3, *95% CI*: 1.1–1.6) had increased complete hepatitis B vaccination rates. Compared to HCWs working in municipal-level hospitals, the odds ratio of complete hepatitis B vaccination increased for HCWs working in county-level hospitals (OR = 1.3, *95% CI*: 1.1–1.6) and in township-level hospitals (OR = 1.6, *95% CI*: 1.4–2.0) (Table 2).

Table 2 presents the results from the multivariate logistic regression analysis of the association between variables and the hepatitis B vaccination coverage rate for HCWs receiving ≥1 dose and ≥ 3 doses.

### Reasons why HCWs did not receive the complete hepatitis B vaccination protocol

Among the 547 HCWs who provided information about the reasons why they were not completely vaccinated, “forgot to receive the follow-up doses” (43%), “having obtained immunity and no need for the follow-up doses” (42%), “too busy to complete the follow-up doses” (40%) and “the number of doses has little effect on vaccine efficacy” (9%) were the top reasons mentioned for incomplete hepatitis B vaccination.

### Reasons why HCWs never received any dose of the hepatitis B vaccine

HCWs who reported not ever receiving any dose of the hepatitis B vaccine (n = 438) were asked why they did not ever undergo vaccination. “Having obtained immunity from work”, “hospitals not providing hepatitis B vaccination activities” and “too busy to be vaccinated” were the main reasons mentioned by 40% (177/438), 40% (173/438) and 32% (140/438) of those respondents, respectively. The most common reason for vaccination refusal among township-level hospital HCWs was hospitals that did not provide hepatitis B vaccination activities (34%), while “having obtained immunity from work” was most commonly reported at both county-level (40%) and municipal-level hospitals (51%) (Table 3). When these 438 HCWs were also asked about their willingness to receive hepatitis B vaccination, only 30% (131/438) indicated they intended to be vaccinated, 30% (133/438) never intended to be vaccinated and 40% (174/438) were undecided.

**Table 3:** Reasons why HCWs never received a hepatitis B vaccine in China (n = 438).

Reasons	Province			Hospital level			Total (n = 438)
	Fujian (n = 124)	Jiangxi (n = 205)	Gansu (n = 109)	Municipal (n = 119)	County (n = 161)	Township (n = 158)	
Having obtained immunity from work	68 (55)	83 (41)	26 (24)	61 (51)	64 (40)	52 (33)	177 (40)
Hospitals do not provide hepatitis B vaccination activities	51 (41)	85 (40)	37 (34)	56 (47)	63 (40)	54 (34)	173 (40)
Too busy to be vaccinated	38 (31)	63 (31)	39 (36)	44 (37)	55 (34)	41 (26)	140 (32)
No recommendation by an official agency	38 (31)	63 (31)	34 (31)	42 (35)	47 (29)	46 (29)	135 (31)
Low risk of infection	39 (32)	51 (25)	24 (22)	39 (33)	27 (17)	48 (30)	114 (26)
Vaccination fees are high	38 (31)	48 (23)	23 (21)	34 (29)	31 (19)	44 (28)	109 (25)
Long and complex vaccination schedule	38 (31)	53 (26)	16 (15)	40 (34)	44 (27)	23 (15)	107 (24)
Healthy enough, no vaccination needed	39 (32)	47 (23)	18 (17)	40 (34)	32 (20)	32 (20)	104 (24)
Worried about efficacy	31 (25)	42 (21)	29 (27)	36 (30)	34 (21)	32 (20)	102 (23)
Worried about side-effects	31 (25)	33 (16)	22 (20)	29 (24)	31 (19)	26 (17)	86 (20)
Don’t know where to be vaccinated	34 (27)	15 (7)	15 (14)	31 (26)	20 (12)	13 (8)	64 (15)
Inconvenience due to distance	29 (23)	16 (8)	11 (10)	26 (22)	18 (11)	12 (8)	56 (13)

## Discussion

The ≥1 dose rate of vaccination among HCWs was 86% in the present study, which was similar to another study (86.4%) of six cities of China [22]. The vaccination coverage rate from our study is similar to other countries (e.g., Brazil 2009, self-reported, 82.4% [30]; Belgium 2004, sero-survey, 84.9% [31]; Germany 2009–2011 sero-survey, 82.0% [32]; Italy 2006, self-reported, 85.3% [33]; Japan 2016, self-reported, 83.7% [34]), but far less than some developed countries (e.g., France 2009, self-reported, 97.0% [12]). Low vaccination coverage among HCWs older than 40 years is partially attributed to the inability of senior staff members to receive the hepatitis B vaccine before 1992 [6].

The complete hepatitis B vaccination rate in this study is relatively low (60%). Surprisingly, the rate in high-risk departments was lower than in low-risk departments, indicating the need to strengthen education targeted to HCWs with a high occupational exposure risk. The complete hepatitis B vaccination coverage among HCWs working in workplaces that only offered a free hepatitis B vaccine without vaccination management program (57%) was not different from HCWs working in workplaces where a free hepatitis B vaccine was unavailable (56%) (*p* = 0.31), whereas hospitals that offered a free hepatitis B vaccine and simultaneously managed HCW vaccination through a vaccine uptake surveillance system with an assessment of anti-HBs status to guide revaccination significantly improved complete vaccination coverage (72%) (*p* <0.01). A recent study reported higher vaccine coverage rates in countries with monitoring systems for HCWs vaccination against hepatitis than in countries without this type of system [32]. In other studies, when hepatitis B vaccination was only recommended or offered for free to HCWs, the main barrier to better compliance with the guidelines was the failure of the employer to ensure that policies are implemented [35–37].

As China considers developing national or subnational hepatitis B vaccination policies for HCWs, clear definitions of who are defined as HCWs should be implemented. The term HCW should include all paid and unpaid persons providing health care or working or training in health-care settings who have reasonably anticipated risks of exposure to infectious materials. Although this study did not sample medical and nursing students, a national HCW hepatitis B vaccination policy should aim to create a culture where blood-borne pathogen screening and vaccination become part of the routine process and where newly hired HBV-infected employees are routinely counselled on the benefits of hepatitis B screening and vaccination. In this study, approximately one-fifth of HCWs were reluctant to be vaccinated for unfounded concerns about the safety and efficacy of vaccines. This finding indicates insufficient hepatitis B vaccination-related knowledge among HCWs. The hepatitis B vaccine has been proven to be safe and effective when administered to infants, children, adolescents and adults by many clinical trials [16, 38, 39]. In addition, only 30% of HCWs who never received any hepatitis B vaccination intended to be vaccinated. This low willingness may be affected by the availability of a free vaccine through their health care facility [40]. Comprehensive measures, such as providing an educational campaign on HBV infection and vaccination knowledge or developing a national or health facility hepatitis B vaccination policy, may be necessary to protect HCWs from HBV infection. This policy should include an implementation and management plan, as well as regularly scheduled occupational medical check-ups for both older HCWs and newly appointed HCWs while they undergo their schooling or training.

This study had some limitations. This study relied on self-reported data that may have introduced recall bias. Several subjects had an obscure memory of their hepatitis B vaccination history, leading to a potential overestimation or underestimation of the hepatitis B vaccination rates. Before the hepatitis B vaccination records of HCWs were recorded and available, a self-reported vaccination history is still feasible for analysis and may be the only method for readily obtaining vaccination histories [41]. In addition, this study only collected information on respondents’ current medical positions, but lacked further information about changes in positions or departments, which may have influenced the accuracy of the corresponding grouping or categorization.

## Conclusions

In conclusion, the complete hepatitis B vaccination rate among HCWs in China and the desire of HCWs to be vaccinated are low. Education campaigns and national hepatitis B vaccination policies targeting HCWs are needed, particularly for older HCWs who may be at a greater risk. A well-planned, well-defined and free nation-wide hepatitis B vaccination policy for HCWs that includes guidance on vaccination management should be prioritized to improve the complete hepatitis B vaccine coverage among HCWs, who are an at-risk group for developing or unknowingly having a chronic HBV infection and subsequently transmitting the hepatitis B infection.

## Supporting information

S1 File This is the English questionaire. (DOCX)

S2 File This is the Chinese questionaire. (DOCX)
