# Supplementary material for: Hepatitis B vaccination coverage among health care workers in China
Source: PLoS One. 2019 May 7;14(5):e0216598. doi: 10.1371/journal.pone.0216598 (PMC6504080; doi:10.1371/journal.pone.0216598)
Supplement: S1 File — (DOCX) [file pone.0216598.s001.docx]

**Survey on Hepatitis B virus status and vaccination coverage among health care workers**

We, staffs of the Chinese Center for Disease Control and Prevention. The aim of the survey is to measure the HBV infection rate and vaccination coverage among Health Care Workers (HCWs), hence, we would like to collect your demographic information as well as information of HBV infection, Hepatitis B related knowledge and hepatitis B vaccination history. The evidence will facilitate the development of hepatitis B vaccine strategies for HCWs in China. We will use the anonymous questionnaire to collect your information to make sure your individual information is confidential, and we promise all information you applied will be properly stored and used only for scientific research reasons, not for any commercial purpose. Please feel free to answer, put a tick in the box if you agree with this statement. Thank you for your support and cooperation!

Province: □ Fujian □Jiangxi □Gansu

Hospital level: □Municipal □County □township

Hospital name: ______________________

**Part A Basic information**

1. Sex: □Female □Male
2. Age: _____years old
3. Working years in profession: _____years
4. Department : _______________
5. Medical occupation: □Physician □Nurse □Technician □Administrator □Cleaner
6. Education: □Postgraduate and above □Undergraduate □Junior college or below
7. Title: □Senior □Intermediate □Junior or low
8. HBV infected numbers in family □Yes □No □Unknown

**Part B Hepatitis B related knowledge**

1. Source of hepatitis B virus infection is from:

□Acute hepatitis B □Chronic hepatitis B □HBV carrier

□Patients with positive HBsAg □Unknown

1. Routes of hepatitis B virus transmission are:

□Daily life and work □Sexual contact □Coughing and sneezing

□Exposure to infectious blood or body fluids □Mosquito bites

□[Vertical transmission](https://en.wikipedia.org/wiki/Vertical_transmission) from mother to child □Gastrointestinal transmission □Unknown

1. Hepatitis B can cause cirrhosis and liver cancer. □Yes □No □Unknown
2. Hepatitis B can reduce risk of HBV infection. □Yes □No □Unknown
3. Currently, hepatitis B can be cured. □Yes □No □Unknown
4. Having finished hepatitis B vaccination means you will never be infected with HBV again

□Yes □No □Unknown

1. Is it necessary to conduct protective antibody sero-test regularly after vaccination?

□Yes □No □Unknown

1. There is no association between hepatitis B efficacy and doses vaccinated.

□Yes □No □Unknown

1. In china, is the hepatitis B vaccination recommended for healthcare workers by the government? □Yes □No □Unknown
2. Have you ever experienced percutaneous or mucosal exposures to blood or body fluids (e.g., semen, saliva, and wound exudates) containing HBV when conducting medical [operatio](file:///D:\\Users\\Administrator\\AppData\\Local\\Youdao\\Dict\\7.5.2.0\\resultui\\dict\\?keyword=operation)ns in hospital? □Yes □No □Unknown
3. Have you ever attended any organized training for hepatitis B control and prevention?

□Yes □No □Unknown

1. Channels you get information about hepatitis B

□Thematic training for hepatitis B control and prevention □Lectures and seminars

□Radio, television or Internet □Books and newspapers □Posters or folding pages

□Government documents (e.g., notices) □CDC □Other_________

**Part C Hepatitis B vaccination history**

1. Have you ever tested hepatitis B related serological marker? □Yes □No □Unknown
2. What’s your present hepatitis B surface antigen (HBsAg) status?

□Positive □Negative □Unknown

1. Have you ever had a hepatitis B vaccination?

□Yes □No( **skip to question 9**) □Unknown

1. When did you receive the vaccination?

□Before working in hospital □After working in hospital □Unknown

1. How many doses did you receive?

□1 dose □2 doses □3 or more doses □Unknown

1. How long from the last dose finished to now?

□less than 3 year □ 3-5 years □6-10 years □more than 10 years

1. What’s the reason for you to receive vaccination?

□Occupational exposure occurring□To protect myself □To protect my family members

□To protect my patients □Hospital entry requirements □Free vaccination is offered

□Other

1. Have you tested your anti-HBs status after finishing the last doses of hepatitis B vaccination?

□Yes □No □Unknown

**(Those who have received hepatitis B vaccination have finished this questionnaire, thank you! )**

1. What are reasons for you not to be vaccinated?( please tick ”√”)

| **Possible Reasons for un-vaccination** | | **Yes** | **no** |
| --- | --- | --- | --- |
| Having gain immunity from work | |  |  |
| Hospitals didn’t provide immunization activities | |  |  |
| Too busy to be vaccinated | |  |  |
| No recommendation by official agency | |  |  |
| Low risk of infection | |  |  |
| Vaccination fees are high | |  |  |
| Long and complex vaccination schedule | |  |  |
| Healthy enough, no vaccination need | |  |  |
| Worried about efficacy | |  |  |
| Worried about side-effect | |  |  |
| Don’t know where to vaccinate | |  |  |
| Inconvenience for far distance | |  |  |
| Other reason ([specify](javascript:;)) | |  | |
|  |  |  |  |

1. Do you intend to be vaccinated? □Yes □No □Not sure or unknown
