# Supplementary material for: Hepatitis B vaccination coverage among health care workers in China
Source: PLoS One. 2019 May 7;14(5):e0216598. doi: 10.1371/journal.pone.0216598 (PMC6504080; doi:10.1371/journal.pone.0216598)
Supplement: S2 File — (DOCX) [file pone.0216598.s002.docx]

**医务人员乙肝疫苗接种率及接种意愿调查问卷**

您好！我们是中国疾病预防控制中心的工作人员，本次调查旨在了解医务人员对乙型肝炎（简称乙肝）相关知识的认知程度、乙肝疫苗接种情况及接种意愿，以便更好地为医务人员乙肝免疫工作提出建议。本次调查采用匿名形式，我们将对您的信息严格保密，您可以放心作答。请您认真阅读每一道题目，根据自己的实际情况在“□”中填写答案或在相应的选项上画“√”，问卷结束后烦请仔细核对有无漏填。衷心感谢您的支持与配合！

调查地区： ①福建 ②江西 ③重庆 □

医院行政级别： ①市级医院 ②县级医院 ③乡级医院 □

医院等级： ①三级医院 ②二级医院 ③一级医院 □

医院名称：______________________

**A．基本信息**

1. 性别：①男 ②女 □
2. 年龄：_____岁
3. 工作年限：_____年
4. 所在科室：_______________
5. 岗位：（见填表说明） □

①医生 ②护士 ③医技人员 ④药剂人员 ⑤行政管理人员 ⑥后勤人员

⑦其他_________

1. 学历：①博士及以上 ②硕士 ③本科 ④大专及以下 □
2. 职称：①高级 ②中级 ③初级 ④其他 □
3. 家庭内是否有乙肝病毒感染者 ①是 ②否 ③不清楚 □

**B．乙肝相关知识**

1. 乙肝的传染源包括哪些？（可多选） □ □ □ □

①急性乙肝患者 ②慢性乙肝患者 ③乙肝病毒携带者 ④乙肝表面抗原（HBsAg）阳性者 ⑤不清楚

1. 您认为以下哪些途径可以传播乙肝病毒？（可多选） □ □ □ □

①日常工作或生活接触 ②性行为 ③打喷嚏、咳嗽或吐痰 ④输血、针刺 ⑤蚊虫叮咬

⑥母婴传播 ⑦消化道传播 ⑤不清楚

1. 您是否同意以下说法？

3.1 乙肝可以导致肝硬化和肝癌 □

①非常同意 ②同意 ③一般 ④不同意 ⑤非常不同意

3.2 目前乙肝可以治愈 □

①非常同意 ②同意 ③一般 ④不同意 ⑤非常不同意

3.3 接种乙肝疫苗可以降低感染乙肝病毒的风险 □

①非常同意 ②同意 ③一般 ④不同意 ⑤非常不同意

1. 接种乙肝疫苗后便不会再感染乙肝病毒 ①是 ②否 ③不清楚 □
2. 接种乙肝疫苗后是否需要定期检测抗体水平 ①是 ②否 ③不清楚 □
3. 乙肝疫苗的免疫效果和接种剂次无关 ①是 ②否 ③不清楚 □
4. 卫生部门是否建议医务人员接种乙肝疫苗 ①是 ②否 ③不清楚 □
5. 您是否发生过职业暴露（破损皮肤接触到病人的血液、体液或被污染的锐利器械损伤）？

①是 ②否 ③不记得 □

1. 您是否参加过医务人员乙肝防治知识培训？ ①是 ②否 ③不清楚 □
2. 您平时通过哪些途径了解乙肝相关信息？（可多选） □ □ □ □

①乙肝防治专题培训 ②知识讲座及研讨会 ③广播、电视和互联网 ④书本、报纸

⑤宣传画、折页等 ⑥政府文件（通知，公告等）⑦疾控中心 ⑧其他_________

**C．乙肝疫苗接种情况**

1. 您是否检测过乙肝相关血清学指标？ ①是 ②否 ③不清楚 □
2. 您目前的乙肝表面抗原（HBsAg）状态为？ ①阳性 ②阴性 ③不清楚 □
3. 您是否接种过乙肝疫苗？ ①是 ②否 ③不记得 □

（**若选①，继续第4题；选②③，请跳至D部分：未接种原因）**

1. 您是何时接种的乙肝疫苗？

①来医院工作之前 ②来医院工作之后 ③其他时间，请注明______ □

1. 您接种最后一针乙肝疫苗距离现在有多长时间？

①<3年 ②3～5年 ③6～10年 ④>10年 □

1. 您接种乙肝疫苗的原因是？（可多选） □ □ □ □

①发生了锐器伤或血液暴露 ②为了保护自己 ③为了保护家人 ④为了保护病人

⑤入职要求接种 ⑥可以免费接种 ⑦其他

1. 您接种过几针乙肝疫苗？ ①1 ②2 ③≥3 ④忘记/不知道 □

**（若选①②，跳至8题，若选③④跳至9题**）

1. 若接种未满3针，原因是？（可多选） □ □ □ □

①感觉已产生免疫力 ②工作忙，没时间接种后续剂次 ③忘记接种后续剂次 ④接种几针对免疫效果影响不大 ⑤其他

1. 您接种疫苗后是否检测过抗体水平 ①是 ②否 ③不记得 □

（**有疫苗接种史者答完此题，调查结束**）

**D. 未接种原因**

1. **您未接种乙肝疫苗，是否是由于以下哪些原因？**（请您根据真实情况，在相应的选项下划“√”）

| **未接种的可能原因** | **非常同意** | **同意** | **一般** | **不同意** | **非常不同意** |
| --- | --- | --- | --- | --- | --- |
| 我感染乙肝的风险不大 |  |  |  |  |  |
| 我身体很健康，不需要接种 |  |  |  |  |  |
| 工作中已产生免疫力，无需再接种 |  |  |  |  |  |
| 平时工作忙，没时间接种 |  |  |  |  |  |
| 乙肝疫苗接种程序繁琐 |  |  |  |  |  |
| 担心乙肝疫苗有副作用 |  |  |  |  |  |
| 担心乙肝疫苗的免疫效果 |  |  |  |  |  |
| 官方没有推荐医务人员接种乙肝疫苗 |  |  |  |  |  |
| 单位不组织接种 |  |  |  |  |  |
| 疫苗接种费用偏高 |  |  |  |  |  |
| 不知道去哪里接种 |  |  |  |  |  |
| 路途远，接种不方便 |  |  |  |  |  |
| 已经感染乙肝病毒 |  |  |  |  |  |
| 其他原因（请注明） |  | | | | |

1. **接种意愿及影响因素**
2. **您是否打算去接种乙肝疫苗？** **①是 ②否** **③视情况而定 ④不知道**  □
3. 您是否同意以下说法？（每个条目均需作答，在对应的选项下划“√”）

| **题目** | **非常**  **同意** | **同意** | **一般** | **不同意** | **非常**  **不同意** |
| --- | --- | --- | --- | --- | --- |
| 医务人员比普通人群更容易感染乙肝病毒 |  |  |  |  |  |
| 病人感染了乙肝病毒，可能传染给医务人员 |  |  |  |  |  |
| 医务人员感染了乙肝病毒，不会传染给病人 |  |  |  |  |  |
| 所有医务人员都应该接种乙肝疫苗 |  |  |  |  |  |
| 仅具有高感染风险的医务人员应该接种乙肝疫苗 |  |  |  |  |  |
| 乙肝不会导致死亡 |  |  |  |  |  |
| 感染了乙肝病毒，工作和生活会受影响 |  |  |  |  |  |
| 乙肝治疗花费巨大 |  |  |  |  |  |
| 接种乙肝疫苗可以保护病人 |  |  |  |  |  |
| 接种乙肝疫苗可以保护家人 |  |  |  |  |  |
| **题目** | **非常**  **同意** | **同意** | **一般** | **不同意** | **非常**  **不同意** |
| 接种乙肝疫苗可以保护同事 |  |  |  |  |  |
| 若乙肝疫苗免费，我会去接种 |  |  |  |  |  |
| 若周围同事接种了，我会去接种 |  |  |  |  |  |
| 若家人建议，我会去接种 |  |  |  |  |  |

**调查结束！感谢您的配合！**

调查员：______________________

调查日期：_____年_____月_____日
